# Effect of Encapsulation Material on Lipid Bioaccessibility and Oxidation during In Vitro Digestion of Black Seed Oil

**DOI:** 10.3390/antiox12010191

**Published:** 2023-01-13

**Authors:** Jon Alberdi-Cedeño, Martha Aichner, Agnes Mistlberger-Reiner, Aimin Shi, Marc Pignitter

**Affiliations:** 1 Department of Physiological Chemistry, Faculty of Chemistry, University of Vienna, 1090 Vienna, Austria; 2 Food Technology, Faculty of Pharmacy, Lascaray Research Center, University of the Basque Country (UPV-EHU), 01006 Vitoria-Gasteiz, Spain; 3 Institute of Food Science and Technology, Chinese Academy of Agricultural Sciences, Key Laboratory of Agro-Products Processing, Ministry of Agriculture and Rural Affairs, Beijing 100081, China

**Keywords:** black seed oil, encapsulation, free oxylipins, bioaccessibility, in vitro digestion

## Abstract

Different encapsulation materials might not only affect lipid hydrolysis but also lipid oxidation during in vitro digestion. Thus, this study aimed to investigate the effect of two commonly used shell materials, starch and gelatin, on the extent of lipolysis and bioaccessibility of the main and some minor lipid compounds, as well as on the oxidative status in encapsulated black seed oil (*Nigella sativa*) during in vitro digestion. The study was carried out using ^1^H nuclear magnetic resonance spectroscopy, liquid chromatography-mass spectrometry and high-performance liquid chromatography-UV. It was shown that starch increased the level of lipid hydrolysis in black seed oil during gastric in vitro digestion, while no differences were observed in the intestinal digestates between starch-encapsulated oil and gelatin-encapsulated oil. Similarly, the bioaccessibility of minor compounds (tocopherols, sterols and thymoquinone) was not influenced by the shell materials. However, regarding lipid oxidation, a 20- and 10-fold rise of free oxylipins was obtained in oils encapsulated by starch and gelatin, respectively, after intestinal in vitro digestion. This study evidenced that gelatin rather than starch should be used for the encapsulation of oils to minimize the digestion-induced formation of bioactive oxylipins.

## 1. Introduction

Vegetable oils are popular for their use as supplements due to their bioactive ingredients. Their oxidation mechanism is a complex subject of great interest and has significant implications in terms of sensory and nutritional quality. As a result of the autoxidation reactions of lipids during storage, processing or even during digestion [1,2,3,4,5,6,7,8], oxidation products, which are related to several diseases, such as heart diseases, cancer, allergenic responses, atherosclerosis, obesity, Parkinson’s disease, and Alzheimer disease [9,10,11,12,13], might be formed. Recently, oxylipins have been shown for the first time to be detected in oxidized corn oil, as well as in virgin flaxseed oil and corn oil subjected to in vitro digestion [4,5,7]. However, the fate of oxylipins during gastric and intestinal digestion is less well known.

In order to slow down the oxidation reactions, extend the shelf life of vegetable oils and reduce the formation of potentially toxic oxidation compounds, two main strategies could be used. On the one hand, natural and/or synthetic antioxidants can be added to the food matrix [5,14] and on the other hand, the lipids could be encapsulated to prevent their direct contact with oxygen, light, moisture and heat [15,16,17]. The latter could be a promising approach to protecting lipids from oxidation reactions, as well as masking off-flavors or colors [18].

Likewise, during in vitro gastrointestinal digestion processes, triacylglycerols contained in vegetable oils undergo hydrolysis. The extent of hydrolysis can be influenced by several factors, such as the proportions of oils/digestive fluids, the composition of digestive juices, the concentration, nature and activity of the enzymes in the juices and the unsaturation degree of the lipids [6,19,20]. In addition, encapsulation of vegetable oils could influence the extent, release and absorption of polyunsaturated fatty acids (PUFAs) and other minor compounds during digestion [21]. In this sense, it should be pointed out that most of the studies in the literature have generally focused on the release of PUFAs during the in vitro digestion of emulsions coated or encapsulated with different shell materials [22]. So far, only a few studies have described the release of PUFAs from encapsulated oils in different shell materials during their in vitro digestion [15,23]. However, these studies only focused on the release of free fatty acids, but as far as we know, none of these studies investigated the overall process of lipolysis in encapsulated oils, giving the molar % of the different acylglycerol structures formed, as well as their bioaccessibility after in vitro digestion. Moreover, there are no studies in the literature addressing the effect of shell material on the formation of potentially toxic compounds and the oxidative stability of edible oils exposed to in vitro digestion.

It can be hypothesized that capsule materials might influence the extent of lipid hydrolysis differently due to their different impacts on emulsification during digestion. As lipolytic products, such as free fatty acids, are more prone to lipid oxidation than triacylglycerols, shell materials might not only differently affect lipid hydrolysis but also oxidation.

In this context, the aim of this study was to analyze the effect of two different shell materials, starch and gelatin, on the extent of lipolysis, the bioaccessibility of main and some minor lipid compounds and on the oxidative status of encapsulated black seed oil (*Nigella sativa*) during in vitro digestion. The aim was achieved using ^1^H nuclear magnetic resonance (^1^H NMR), liquid chromatography-mass spectrometry (LC-MS) and high-performance liquid chromatography-UV (HPLC-UV). It can be expected that free oxylipins might not only be detected in vegetable oils after in vitro digestion by NMR, as described previously [4,5], but also during digestion by LC-MS to investigate the fate of oxylipins after the gastric and intestinal phases. Black seed oil (*Nigella sativa*) was selected because of its high therapeutic value and widespread use as a food supplement. The results give a holistic view of the different reactions that take place during in vitro digestion, focusing not only on one compound but on several markers.

## 2. Materials and Methods

### 2.1. Samples

The study was carried out with commercially encapsulated black seed oil (*Nigella sativa*) provided by Goerlich Pharma, Edling, Germany. Each capsule contained 500 mg of Egyptian black seed oil encapsulated in either a soft starch capsule (BS1) or a soft gelatin capsule (BS2). The soft starch capsule consisted of modified corn starch (70.6 mg), glycerol (E422) (80.2 mg), carrageenan (E407) (28.2 mg), sodium carbonate (anhydrous) (0.1 mg) and water (14.9 mg), while the soft gelatin capsule was composed of gelatin (bovine) (129.3 mg), glycerol (E422) (66.0 mg) and water (14.7 mg). The composition of this oil in molar percentages of linoleic (L), oleic (O) and saturated (S) acyl groups was 55.3 ± 0.71%, 27.8 ± 0.75% and 16.9 ± 0.09%, respectively, as determined by ^1^H NMR and reported in previous studies [4,5,6].

### 2.2. Chemicals and Materials

Sodium hydroxide, potassium chloride, potassium dihydrogen phosphate, magnesium chloride hexahydrate, calcium chloride dihydrate, albumin, hydrochloric acid, dichloromethane, acetic acid and acetic acid ethyl ester were purchased from Carl Roth GmbH & CO., Karlsruhe, Germany. Sodium chloride, urea, uric acid, potassium thiocyanate, pancreatin from porcine pancreas, pepsin from porcine gastric mucosa, α-amylase from *Aspergillus oryzae*, sodium sulfate, sodium hydrogen carbonate, mucin, lipase from porcine pancreas, D-glucose, bile bovine, ammonium chloride, Amano lipase A from Aspergillus niger, α-tocopherol, γ-tocopherol and δ-tocopherol were obtained from Sigma Aldrich, St. Louis, MO, USA. Acetonitrile (LC-MS grade) and methanol (LC-MS grade) were acquired from Avantor/VWR International Inc. (Radnor, PA, USA). Chloroform D + 0.03% TMS was purchased from Eurisotop, Cambridge Isotope Laboratory Inc., Andover, USA. β-tocotrienol was acquired from Biomol GmbH, Hamburg, Germany. Sodium Dihydrogen Orthophosphate Dihydrate was obtained from Fisher Chemical Co., Fair lawn, NJ, USA and rac-Tocol was purchased from Abcam.

### 2.3. In Vitro Digestion of the Capsules

An in vitro digestion was employed based on a semi-static procedure that mimics digestive processes occurring in the mouth, stomach and duodenum by sequentially adding simulated digestive juices (saliva, gastric juice, duodenal juice and bile juice) to the sample, while incubated at 37 ± 2 °C and rotated head-over-heels throughout the duration of the experiment. This in vitro procedure was initially developed by Versantvoort et al. (2005) [24] and slightly modified by Nieva-Echervarría et al. (2016) [20] to reach a higher degree of lipolysis, similar to that occurring in vivo. The capsules containing 500 mg of oil were subjected in triplicate to in vitro gastrointestinal digestion. In short, 6 mL of simulated saliva juice were added to each sample and incubated on the analog rotator RS-RR 5 (Phoenix Instrument, Garbsen, Germany) in the oven at 37 ± 2 °C for 5 min. A total of 12 mL of gastric juice was added to the tubes afterwards. After one hour, the pH was measured and adjusted to 2–3 with NaOH (10 M). One hour later, in vitro gastric digestion was completed. The pH was measured and 2 mL NaHCO_3_ (1 M), 12 mL simulated duodenal juice and 6 mL simulated bile juice were added. The pH was adjusted to 6–7 and the tubes were placed in the oven on the rotator for another 4 h. After the in vitro digestion and in order to stabilize the samples, the digestates were stored at −80 °C until extraction of the lipids. The composition of the juices is shown in the Supplementary Material. All samples were analyzed in triplicate after oral, gastric and intestinal in vitro digestion, as indicated in the results.

### 2.4. Extraction of the Lipids from the Digestates

After in vitro digestion, the lipids of the digestates were extracted with dichloromethane, as in previous studies [4,5,6]. Briefly, a six-fold extraction was performed, adding 15 mL dichloromethane to the samples, centrifuging them at 14,000 rpm for 7 min, taking the lower, non-polar phase, unifying the extracts and evaporating the solvent to obtain the digested lipids. The samples were stored at −40 °C under argon until further analysis.

### 2.5. ^1^H NMR Analysis

The ^1^H NMR spectra of the oils (BS1 and BS2) and of the lipid extracts of their digestates after gastric in vitro digestion (G-BS1, G-BS2) and intestinal in vitro digestion (I-BS1 and I-BS2) were acquired in triplicate using an Avance 400 spectrometer (Bruker, Billerica, MA, USA) operating at 400 MHz. For this purpose, 200 μL samples were dissolved in 400 μL of deuterated chloroform, which contained tetramethylsilane (TMS) as an internal reference. The acquisition conditions were the same as those used in previous studies [4]. The ^1^H NMR spectra were plotted at a fixed value of absolute intensity to be valid for comparative purposes using the MestreNova program (Mestrelab Research, Santiago de Compostela, Spain).

The identification of the acylglycerol structures, acyl groups, fatty acid composition, sterols and oxidation products was carried out on the basis of the bibliographic data [4,5,6,25,26]. The quantification of the molar percentage of each kind of acylglycerol structure was determined using the equations in previous studies [27]. The molar percentages of different acyl groups and fatty acids, in relation to the total moles of all kinds of fatty acids plus acyl groups (AG+FA), were estimated as in a previous study [6]. Likewise, the concentration of sterols and lipid oxidation products was analyzed based on their ^1^H NMR spectral signals using the general equation as previously published [6].

### 2.6. Nanoparticle Tracking Analysis

To characterize the oil droplets within the digestates, the particle concentration, particle size, and size distribution of all particles, including the oil droplets, were determined. Nanoparticle tracking analysis (NTA) was performed with ZetaView PMX 120-Z equipped with a 520 nm laser (Particle Metrix, Inning am Ammersee, Germany). Before the NTA measurements, the black seed oil was digested, as described above. Samples were analyzed after oral, gastric and intestinal in vitro digestion. The instrument settings were optimized to detect particles with a mean size of around 200 nm, and the following parameters were chosen: sensitivity: 60, shutter: 150, frame rate: 15; post-acquisition parameters: min. brightness: 20, min. area: 10, max. area: 10,000, tracelength: 30. All measurements were performed at a flow cell temperature of 22 °C, and the samples were diluted in water to reach about 100–200 particles per frame. For each replicate, 3 measurements were performed, each measuring 11 positions. At least 1000 particles per replicate were measured and included in the analysis for size determination. The data analysis was carried out using ZetaView software, version 8.05.14 SP7.

### 2.7. Sample Preparation for LC-MS Analysis

A solid phase extraction (SPE) was performed to extract the free oxylipins from the black seed oil or from the lipid extract of the digestates, based on a previous study [28], with some modifications. In short, the SPE cartridge, Strata™-x 33 μm Polymeric Reversed Phase, 30 mg/1 mL tube (Phenomenex, Torrance, CA, USA) was washed with 1 mL ethyl acetate and 2 × 1 mL methanol and conditioned with 2 × 1 mL buffer (5% methanol in water with 0.1% acetic acid). A total of 50 mg of oil or lipid extract of digestates was diluted in 2 mL ethyl acetate and loaded onto the cartridge. Then, the cartridge was washed twice with 1 mL buffer. After drying the cartridge 20 min under vacuum, the free oxylipins were eluted from the cartridge with 1 mL methanol and 3 × 1 mL ethyl acetate. The samples were dried under nitrogen and reconstituted with 300 μL of methanol. After filtration with syringe filters (Phenomenex, Phenex™-NY 15 mm, 0.2 u), the samples were analyzed using LC-MS.

### 2.8. LC-MS Analysis of Free Oxylipins

To determine the free oxylipins present in the oil and digestates, the samples (20 μL) were injected onto an LC-20 system with an LCMS-8040 detector (Shimadzu, Korneuburg, Austria). The free oxylipins were separated by the LC-20 using a C12 column (Synergi 4 μm Max-RP 80 Å, 150 × 2 mm, Phenomenex, Torrance, CA, USA) at 25 °C. The mobile phase was H_2_O with 0.1% acetic acid (solvent A) and acetonitrile/methanol (80/15) (solvent B). The LC gradient was 25% solvent B for the first minute and increased to 30% until 1.5 min; from 30% to 53% until 10 min; from 53% to 68% until 19.5 min; 68% to 95% until 24.5 min; hold it until 27 min; 95% to 25% until 27.1 min; and hold it until 30.5 min. The flow rate was 0.3 mL/min. The following ESI ion source settings were applied: nebulizing flow 3 L/min, drying gas flow 10 L/min, desolvation line temperature 150 °C and heat block temperature 350 °C. Argon with different collision energies was used for MS/MS (Table S2). For the identification, the mass spectrometric analysis for each free oxylipin was performed in the multiple reaction monitoring (MRM) mode on the ESI triple quadrupole mass spectrometer in negative mode, according to a previous study [28]. For semi-quantification, the abundance of free oxylipins was determined based on the area under the curve of the corresponding MRM transition. The free oxylipins with the corresponding transitions, collision energies and retention times are listed in Table S2. Data analysis was performed with Labsolution software version 5.99 SP2.

### 2.9. Determination and Quantification of Tocols by HPLC-UV

Tocols were analyzed as described previously [8]. Briefly, a 100 mg sample (oil or lipid extract of the digestates) was dissolved in 300 µL 2-propanol. A total of 1 µL internal standard was added to the sample before the sample was filtered with syringe filters (Phenomenex, Phenex™-NY 15 mm, 0.2 u). The samples were analyzed by the LC-20 (Shimadzu, Korneuburg, Austria) equipped with a C18 column (C18 Kinetex, 5 µm, EVO C18, 150 mm × 2.6 mm) at 10 °C and a photodiode array detector (SPD-M30A, Shimadzu, Korneuburg, Austria). The flow rate was 0.5 mL/min. The mobile phase was water (solvent A) and methanol (solvent B). The gradient was: 95–100% from 0 min to 4 min, 100% from 4 min to 14 min, 100–95% from 14 min to 16 min and 95% from 16 min to 18 min. The tocols were measured at a wavelength of 294 nm. Standard curves of α-tocopherol and γ-tocopherol with the linear range concentrations from 0.98–62.5 μg/mL and from 7.81–500 μg/mL for β-tocotrienol were used to quantify the tocols in the samples. The limit of quantification was determined by a signal to noise ratio of 10. The recovery of tocols for fresh black seed oil, digestate after gastric in vitro digestion and digestate after intestinal in vitro digestion were 110 ± 11%, 71 ± 9% and 79 ± 12%, respectively.

### 2.10. Statistical Analysis

Statistically significant differences were determined by one-way ANOVA or two-way ANOVA followed by Tukey’s post-hoc test or by Student’s *t*-test with GraphPad Prism 9.1.2, as indicated in the results section. The results were considered to be significant with a *p*-value lower than 0.05 (*p* < 0.05). For each experiment, at least 3 independent samples were measured.

## 3. Results and Discussion

### 3.1. The Extent of Lipid Hydrolysis during In Vitro Digestion

In order to evaluate the impact of in vitro digestion and capsule materials on the degree of lipid hydrolysis in black seed oil, the lipolytic products, diacylglycerols (1,2-DG and 1,3-DG) and monoacylglycerols (1-MG and 2-MG), derived from the hydrolysis of triacylglycerols (TG), were quantified by ^1^H NMR (Figure S1). Moreover, lipid bioaccessibility, described by the ratio between the concentration of these absorbable species and all fatty acids (FA) plus acyl groups (AG) present in the corresponding digestate, was calculated as in previous studies [4,5,6] (see Table 1).

Vegetable oils are complex mixtures represented mainly by triacylglycerols, with around 99% [4,5,6]. However, as can be observed in Table 1, BS1 and BS2 oils were already slightly hydrolyzed, showing a lower percentage of TG and the presence of some other acylglycerol species, such as 1,2-DG, 1-MG and 2-MG. Furthermore, as can be observed in Table 1, the molar percentage of the acylglycerol species varied slightly between the BS1 and BS2 oils. In more detail, BS1 presents lower TG% but higher amounts of 1,2-DG, 2-MG and Gol, resulting in statistically significantly higher lipid bioaccessibility (LB (%), *p* > 0.05) when compared with BS2.

The triacylglycerol hydrolysis pattern of encapsulated black seed oils after intestinal in vitro digestion (I-BS1 and I-BS2) was very similar, with the presence and the same concentration of diacylglycerols, monoacylglycerols and glycerol in both digestates (Table 1). No significant (*p* > 0.05) differences were found among the molar percentages of the different acylglycerols present in the lipid extracts of encapsulated (I-BS1 and I-BS2) oil digestates, nor regarding the lipid bioaccessibility (LB%) after intestinal in vitro digestion. Furthermore, the percentages of TG decreased from 76.9% (BS1) and 82.7% (BS2), near to 24–25%, reaching triacylglycerols’ transformation of around 70%. The molar percentages of the lipolytic products of TG, 1,2-DG, 1,3-DG, 1-MG and 2-MG, were 14–16%, 4%, 5–6% and 17–18%, respectively (Table 1). Likewise, the lipid bioaccessibility parameter showed values of 60–63%. These results are similar to those reported in previous studies on sunflower and corn oils rich in omega-6 acyl groups after in vitro digestion [4,29]. These findings showed that the encapsulation of black seed oil with either starch or gelatin did not have any effect on the extent of lipolysis after intestinal in vitro digestion.

However, significant differences (*p* < 0.05) related to the capsule material were identified in the digestates after gastric in vitro digestion. Black seed oil encapsulated in starch material showed higher hydrolysis, with a decrease in the molar percentage of TG of 34.3%, compared with black seed oil encapsulated in gelatin, revealing a decline of only 18.5%. Moreover, 2-MG and Gol were 3-fold and almost 2-fold higher, respectively, in G-BS1 than in G-BS2. Therefore, lipid bioaccessibility after gastric in vitro digestion was statistically significantly higher for the oil encapsulated in starch material (37%) than for the oil encapsulated in gelatin (21%), which means that during gastric in vitro digestion, the shell material had an effect on the lipolysis of black seed oil. This effect could be due to the presence of α-amylase in the oral juices, which can start hydrolyzing starch material enhancing, therefore, the access of the lipase added with the gastric juices. These results are in agreement with previous studies [15], which showed that the release of FFA during oil digestion was higher when the oil was encapsulated in gum material than in protein. This effect could be explained by the finding that the shell materials affected the interfacial behavior and might interfere with the diffusion and adsorption of lipase to the oil droplets, as explained by Timilsena and coworkers (2017) [15]. Furthermore, Mun and coworkers (2007) [22] reported that the extent and rate of lipid hydrolysis depends on the interfacial composition. Therefore, shell materials or surfactants with much higher surface activity than lipase could create an interfacial layer around the oil droplets that would prevent the enzyme from approaching the triacylglycerols [22]. Considering this hypothesis, it could be suggested that gelatin might have a higher surface activity than starch and lipase, so the lipolytic enzyme cannot easily access the triacylglycerols in the oil.

Another explanation for the difference in lipid hydrolysis might be based on the oil droplet size within the oil-in-water-emulsion that is formed during digestion. When the oil droplets are smaller, the total surface area available for the adsorption of lipase becomes larger; therefore, enzymatic digestion becomes faster [15]. Since the capsule material could influence the oil droplet size or emulsion stability during digestion [15], this could affect lipid hydrolysis. To test this hypothesis, digestates of black seed oil encapsulated in starch and gelatin after oral (O-BS1 and O-BS2), gastric (G-BS1 and G-BS2), and intestinal (I-BS1 and I-BS2) in vitro digestion were analyzed using nanoparticle-tracking analysis. The concentration and size distribution of particles, including the oil droplets, in the size range of about 50 to 1000 nm were assessed.

**Figure 1:**
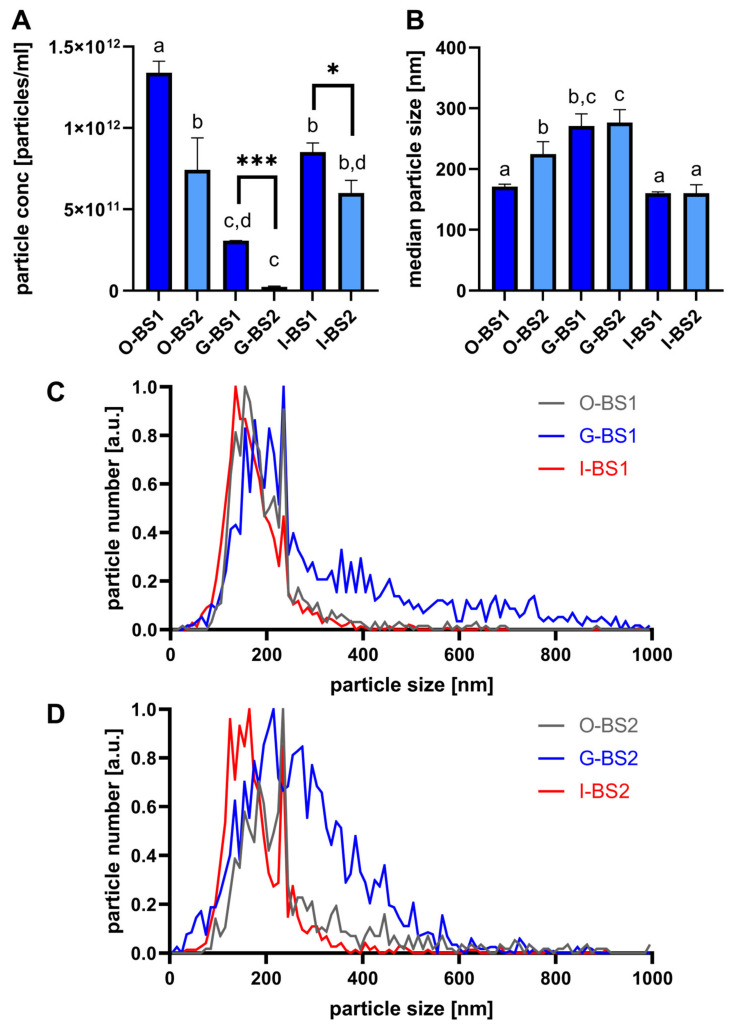
Particle concentration (particles/mL) (**A**) and median particle size (nm) (**B**) in the digestates of starch-encapsulated black seed oil (BS1) or gelatin-encapsulated black seed oil (BS2) after oral (O-BS), gastric (G-BS), or intestinal (I-BS) in vitro digestion. Particle size distribution in the different digestates of BS1 (**C**) and BS2 (**D**), respectively. Different letters indicate statistically significant differences among the samples analyzed by one-way ANOVA (*p* < 0.05). Asterisks indicate statistically significant differences between the indicated samples analyzed by student’s *t*-test (*** = *p* < 0.001, * = *p* < 0.05).

After oral in vitro digestion, the particle concentration in the digestate of the starch-encapsulated black seed oil (O-BS1) was almost twice as high as that in the gelatin-encapsulated oil (O-BS2) (Figure 1A). Additionally, the particles from O-BS1 were smaller (Figure 1B–D) compared to those from O-BS2, suggesting that after oral in vitro digestion, there were more and smaller oil droplets present in O-BS1, which could provide a greater surface area for lipase adsorption and lipid hydrolysis during the following gastric in vitro digestion. After gastric in vitro digestion, the particle concentration was reduced and the median particle size was increased in both samples compared to each sample before this digestion step, as visible in Figure 1A,B. This could mean that during gastric in vitro digestion, oil droplets merged and formed bigger and fewer droplets. When comparing G-BS1 to G-BS2, the median particle size did not differ significantly (Figure 1B), but the size distribution profile clearly showed that much bigger particles were present in G-BS2 (see Figure 1C,D).

In addition, the particle concentration was still higher in G-BS1 when directly compared to G-BS2 (Student’s *t*-test, *p* < 0.001). Gelatin might not favor emulsification of the oil at the gastric stage, leading to reduced hydrolysis due to limited access of lipase to the triacylglycerols. The degree of emulsification might be the determining factor for the slower digestion of gelatin-encapsulated oil. Starch seemed to show suitable emulsifier properties during simulated gastric digestion. Therefore, the difference in the particle concentration, as well as the different size distribution profiles, suggests that the starch-encapsulated oil contained more and smaller particles compared to the gelatin-encapsulated oil after gastric in vitro digestion. Finally, after intestinal in vitro digestion, the median particle size decreased in both samples to a similar level (Figure 1B), which could also be observed for the size distribution profiles (Figure 1C,D). In addition, the particle concentration increased with decreasing size (Figure 1A–D), meaning that the bigger particles formed during gastric digestion were digested into smaller and more homogenous particles during intestinal in vitro digestion. Nevertheless, I-BS1 still contained more particles directly compared to I-BS2 (student’s *t*-test, *p* < 0.05, Figure 1A).

These data support the hypothesis that a higher surface area of oil droplets was available during oral and gastric in vitro digestion of starch-encapsulated oil compared to gelatin-encapsulated oil. This might explain the increased levels of lipid hydrolysis for starch-encapsulated oil after gastric in vitro digestion (G-BS1). Therefore, it seems plausible that the encapsulation material influenced the digestive processes via the oil droplet size. It must be highlighted that this is the first time that the effect of oil encapsulation on the progress of lipid hydrolysis during in vitro digestion has been addressed.

### 3.2. Assessment of Lipid Oxidation of Encapsulated Black Seed Oil during In Vitro Digestion by ^1^H NMR

#### 3.2.1. Changes in the Molar Percentages (%) of Black Seed Oil Acyl Groups and Fatty Acids

**Figure 2:**
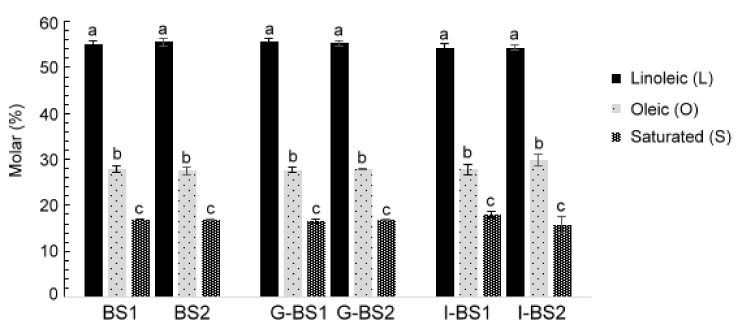
Molar percentage of linoleic, oleic and saturated fatty acids (FA) plus acyl groups (AG) (FA+AG) in relation to the total moles of all kinds of AG and FA (FA+AG) in black seed oils 1 and 2 (BS1 and BS2) and in the digestates of these oils after gastric (G-BS1 and G-BS2) and intestinal (I-BS1 and I-BS2) digestion. Different letters indicate statistically significant differences among the samples analyzed by one-way ANOVA (*p* < 0.05).

During in vitro digestion, the changes in the molar percentages of acyl groups might be more or less marked, depending on their unsaturation degree and the presence or absence of prooxidant or antioxidant compounds [4,30,31]. Figure 2 depicts the molar percentages (%) of different acyl groups in relation to the total moles of AG+FA present in black seed oils before in vitro digestion (BS1 and BS2) and in encapsulated black seed oil samples after gastric and intestinal in vitro digestion (G-BS1, I-BS1, G-BS2 and I-BS2). No significant differences (*p* > 0.05) were found in the molar percentages of linoleic (L), oleic (O) and saturated (S) acyl groups and fatty acids after gastric and intestinal in vitro digestion compared to the oils before digestion. Moreover, no significant changes were observed due to capsule material. The lack of differences indicates that if oxidation has taken place, this has not produced variations in the concentration of the different kinds of acyl groups + fatty acids at a level detectable by means of ^1^H NMR spectroscopy.

#### 3.2.2. Formation of n-Alkanals

**Figure 3:**
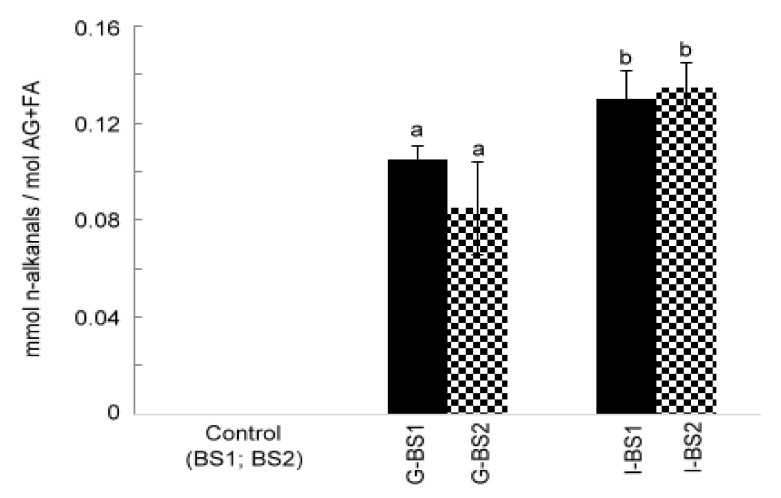
Concentration of n-alkanals expressed as mmol per mol of AG+FA in black seed oils 1 and 2 (BS1 and BS2) and in the digestates of these oils after gastric (G-BS1 and G-BS2) and intestinal (I-BS1 and I-BS2) in vitro digestion. Different letters indicate statistically significant differences among the samples analyzed by one-way ANOVA (*p* < 0.05).

As has been described in several studies before, under in vitro digestion conditions, vegetable oil might undergo degradation and therefore some oxidation compounds might be generated [4,5,6,8]. It is well known that during the lipid oxidation process, lipid hydroperoxides are formed. These primary oxidation compounds are intermediate compounds in the oxidation process and can evolve to form secondary oxidation compounds, such as aldehydes. During the in vitro digestion of black seed oil, n-alkanals were detected, indicating that oxidation took place (Figure 3). The main increase in the concentration of n-alkanals occurred after gastric in vitro digestion. Moreover, the lipid extract of the digestates of all samples after intestinal in vitro digestion presented a higher oxidation degree than those after gastric in vitro digestion, showing a statistically significant (*p* < 0.05) increase in the concentration of n-alkanals.

### 3.3. Assessment of Lipid Oxidation of Encapsulated Black Seed Oil during In Vitro Digestion by LC-MS: Formation and Detection of Free Oxylipins

With the purpose of corroborating and reinforcing the results obtained by ^1^H NMR and to obtain an overall view of the oxidation process during in vitro digestion, LC-MS was used, paying attention to the formation of several free oxylipins derived from the oxidation of omega-6 acyl fatty acids, since these are the main ones in black seed oil. These oxidation compounds can be defined as a set of structurally very diverse molecules derived from the oxidation of polyunsaturated fatty acids (PUFAs) and possessing at least one oxygenated group in their structure, in addition to the carboxylic group. It should be noted that these free oxylipins can be absorbed after digestion and play a key role in diseases such as cancer, Alzheimer’s, Parkinson’s, acute respiratory distress syndrome (ARDS), circulatory shock, disseminated intravascular coagulation and multiple organ failure, among others [12].

Both black seed oils, BS1 and BS2, presented the same pattern and amount of oxylipins, which indicates that these oils were already slightly oxidized. Among the oxylipins detected in the fresh oils and in the digestates, there were 9-hydroperoxy and 13-hydroperoxy octadecadienoic acids (9-HpODE and 13-HpODE), 9-hydroxy and 13-hydroxy octadecadienoic acids (9-HODE and 13-HODE), 9-oxo and 13-oxo octadecadienoic acids (9-oxoODE and 13-oxoODE), 9,10-epoxy and 12,13-epoxy octadecenoic acids, (9(10)-EpOME and 12(13)-EpOME), 12,13-dihydroxy and 9,10-dihyroxy octadecenoic acids, (9,10-DiHOME and 12,13-DiHOME), and 9,10,13-trihydroxy and 9,12,13-trihydroxy octadecenoic acids (9,10,13-TriHOME and 9,12,13-TriHOME). Table 2 shows the abundances of different classes of oxylipins, such as monohydroperoxydienes (HpODE), monohydroxydienes (HODE), monoketodienes (oxoODE), monoepoxymonoenes (EpOME), dihydroxymonoenes (DiHOME) and trihydroxymonoenes (TriHOME), in the different black seed oils and their digestates.

As mentioned above, both fresh black seed oils (BS1 and BS2) already showed a basal oxidation level (Table 2). Although no significant changes were observed in the acyl groups + fatty acid concentrations before digestion and after gastric and intestinal in vitro digestions, an increase in the oxidation level with respect to their control was observed in the lipid extract of all samples. However, the abundance of the oxidation compounds formed varied depending on the digestion phase and the shell material. During gastric in vitro digestion, the highest increase of oxylipins was observed in G-BS1, with HpODE and HODE forming at the highest level. G-BS1 showed a 4-fold higher level of oxylipins than G-BS2, suggesting that the oxidation was more pronounced in the oil encapsulated in starch than in gelatin. This could be explained, on the one hand, by the fact that BS1 contained an already higher concentration of FFA than BS2 (Table 1). Moreover, as commented before, G-BS1 digestate presented higher hydrolysis levels, therefore showing a higher concentration of FFA than G-BS2. This higher concentration in FFA could make the digestates more prone to oxidation, since FFAs are more susceptible to oxidation than triacylglycerols [32]. On the other hand, G-BS1 presented a higher surface area for oil droplets than G-BS2. This finding might favor the presence of more FFA at the surface where oxidation is considered to occur.

Likewise, the formation of oxylipins increased after intestinal digestion in both I-BS1 and I-BS2 digestates. However, differences in the level of oxidation were found between the digestates. Thus, I-BS1 presented a 2-fold higher oxidation level than I-BS2, corroborating that the lipids of oil encapsulated in starch capsules are more susceptible to oxidation. The presence of some of these oxylipins, such as HpODE, HODE, oxoODE and EpOME, have been described previously in the digestates of corn oil enriched with α-tocopherol by ^1^H NMR [4]. However, as far as we know, this is the first time that LC-MS has been used for the identification and semi-quantification of free oxylipins in oils subjected to in vitro digestion. Moreover, no studies have demonstrated so far the effect of capsule materials on the oxidative stability of edible oils during in vitro digestion, nor that gelatin could be a superior capsule material in comparison with starch to limit digestion-induced oxidative deterioration of lipids.

Overall, it should be pointed out that gelatin capsules showed better protection against oxidation, which is of great importance not only for food safety and quality but also for human health. The enhanced formation of free oxylipins during the in vitro digestion of oils encapsulated in starch material might lead to higher bioaccessibility and availability for absorption, promoting pathophysiological conditions.

### 3.4. In Vitro Assessment of the Bioaccessibility of Some Minor Compounds Present in Black Seed Oil

As is well known, during in vitro digestion, not only the main components present in the oils are degraded but also the minor compounds (tocopherols, sterols, terpenes) might be degraded or transformed [5,6,31,32]. Black seed oils contain minor compounds such as tocopherols, tocotrienols, sterols, terpenes and thymoquinone [26,33,34]. This study focused on the degradation and bioaccessibility of tocols (γ-tocopherol, α-tocopherol, β-tocotrienol), sterols (Δ7-avenasterol, cholestanol and esters of cycloartenol), and thymoquinone, since they have important biological activities [35,36,37,38,39]. Some of them give signals in the ^1^H NMR spectra, which do not overlap with any others, enabling their quantification with this method [6,25,26], and others, such as tocols, can be quantified by means of HPLC-UV [8], allowing the estimation of their evolution and bioaccessibility during in vitro digestion. The bioaccessibility of these compounds was calculated as the ratio between their concentration in the digestate (C_D_) and their concentration in the oil before digestion (C_O_); bioaccessibility = [C_D_]/[C_O_].

#### 3.4.1. In Vitro Bioaccessibility of Tocols Present in Black Seed Oil

As presented in Table 3, the main tocols in BS1 were γ-tocopherol and β-tocotrienol followed by α-tocopherol, while BS2 showed lower concentrations of both γ-tocopherol and α-tocopherol. During in vitro digestion, these compounds can be subjected to degradation [4,5,30,31,32]. Their bioaccessibility depends mainly on its initial concentration in foods, the type of fluids used and/or the conditions during digestion, among other factors [4,5,30,31].

The bioaccessibility of α-tocopherol after gastric in vitro digestion was more pronounced the higher the concentration in the fresh oil, showing values around 70% for G-BS1 and 35% for BS2. However, this tocol was not detectable in either intestinal digestate (I-BS1 and I-BS2), suggesting that it would not be able to be absorbed. Regarding the γ-tocopherol after intestinal digestion, a bioaccessibility of 63% and 33% was observed for I-BS1 and I-BS2, respectively, again indicating that a higher concentration of tocols in the fresh oil led to a lower degradation and a higher bioaccessibility of tocols. This tendency is in agreement with a recently published study [4]. Likewise, the β-tocotrienol concentration remained almost stable during the gastric in vitro digestion, although it was partially degraded after intestinal in vitro digestion, indicating that up to around 67% (I-BS1) and 57% (I-BS2) of it remained in the digestates without degrading and being available for absorption. Overall, tocols present in the black seed oil encapsulated in gelatin (BS2) showed a higher degradation rate than BS1, although as above-commented, BS2 was less oxidized during in vitro digestion. This could be explained by the fact that mainly during gastric digestion, BS2 suffered from a low degree of hydrolysis with the release of low amounts of FFA. The low abundance of FFA at the surface might facilitate the passage of oxygen. Thus, the oxygen could have more access to the oil droplets where the lipophilic tocols might have been located, thereby promoting their oxidation.

#### 3.4.2. In Vitro Bioaccessibility of Other Minor Compounds Present in Black Seed Oil

Using ^1^H NMR Δ7-avenasterol, sitostanol, and the esters of cycloartenol and thymoquinone were quantified in BS1 and BS2 and in their corresponding digestates. Sterols were quantified using the area of the signals at 0.54 ppm (Δ7-avenasterol), at 0.58 ppm (esters of cyloartenol) and at 0.65 ppm (sitostanol) and thymoquinone at 6.58 ppm based on the assignment given in previous studies [7,25,26]. As can be observed in Table 3, the concentrations of some sterols and thymoquinone were slightly higher in BS2 than in BS1, showing statistically significant differences for Δ7-avenasterol and thymoquinone. The concentration of the esters of cycloartenol remained unchanged during in vitro digestion, which means that this sterol is fully bioaccessible after both gastric and intestinal in vitro digestion. These results are in accordance with those shown in a previous study during the in vitro digestion of olive oil [6]. However, both Δ7-avenasterol and sitostanol, as well as thymoquinone, were degraded during the in vitro digestion of BS1 and BS2. As Table 3 shows, the concentration of Δ7-avenasterol remained stable during gastric digestion in both the BS1 and BS2 digestates. However, a significant degradation was observed for this compound after intestinal digestion, I-BS1 and I-BS2, resulting in a bioaccessibility of around 70%. Likewise, significant changes in the thymoquinone concentration were observed after gastric and intestinal digestion, with around 60% remaining bioaccessible after in vitro digestion. This fact is of great importance since these compounds have been related to several health benefits [35,36]. However, as can be observed in Table 3, sitostanol is totally degraded after intestinal digestion. Thus, overall, no significant differences were observed in the degradation or bioaccessibility of the minor compounds due to the capsule material. This section may be divided into subheadings. It should provide a concise and precise description of the experimental results and their interpretation, as well as the experimental conclusions that can be drawn.

## 4. Conclusions

This study evidenced for the first time that capsule material has an impact on the extent of lipolysis and oxidative status of encapsulated black seed oil during in vitro digestion processes. An increase in the level of lipid hydrolysis for the starch-encapsulated oil after gastric in vitro digestion compared to the gelatin-encapsulated oil was observed, although no significant differences were found after intestinal in vitro digestion. On the one hand, this effect could be explained by the fact that gelatin might have a higher surface activity than starch and lipase during gastric in vitro digestion. Therefore, the lipolytic enzyme cannot easily access the triacylglycerols in the oil since gelatin could create an interfacial layer around the oil droplets, preventing the enzyme from approaching the triacylglycerols. On the other hand, the difference in lipid hydrolysis might result from the influence of the encapsulation material on the oil droplet size and, therefore, the surface area accessible to digestive enzymes. The latter hypothesis was supported by the analysis of the lipid droplets, which proved to be smaller and had a higher concentration in the digestates of the starch-encapsulated oil. Likewise, it has been shown for the first time that during in vitro digestion of encapsulated black seed oil, gelatin capsules showed better protection against oxidation, which is of great importance not only for food safety and quality but also for human health. The enhanced formation of free oxylipins during in vitro digestion of oils encapsulated in starch material might lead to higher bioaccessibility and absorption, promoting pathophysiological conditions. Overall, no significant differences were observed in the degradation or bioaccessibility of minor compounds naturally present in the oil due to capsule material. Therefore, this study evidenced that gelatin could be a better option than starch for the encapsulation and stabilization of edible oils. It not only provides novel mechanistic results regarding the formation of potentially toxic oxylipins in food supplements during in vitro digestion, thereby advancing science in this field but also clear recommendations for the food supplement industry regarding the use of capsule materials to limit the extent of lipid oxidation.

## Figures and Tables

**Table 1:** Molar percentages of triacyglycerols (TG%), diacylglycerols (1,2-DG% and 1,3-DG%), monoacylglycerols (1-MG% and 2-MG%) and glycerol (Gol%) in relation to the total number of glyceryl structures and lipid bioaccessibility (LB %) in black seed oil samples (BS1 and BS2), encapsulated black seed oil samples after gastric (G-BS1, G-BS2) and intestinal (I-BS1, I-BS2) in vitro digestion.

	Acylglycerol Species (Molar %)						LB (%)
Samples	TG%	1,2-DG%	1,3%-DG%	1-MG%	2-MG%	Gol%	
BS1	76.9 ± 0.19 a	5.29 ± 0.11 a	-	1.50 ± 0.04 a	0.17 ± 0.01 a	16.2 ± 0.34 a	19.6 ± 0.26 a
G-BS1	42.6 ± 1.10 b	27.9 ± 0.36 b	2.40 ± 0.29 a	0.84 ± 0.04 b	6.34 ± 0.39 b	20.0 ± 0.64 b	37.2 ± 1.03 b
I-BS1	24.0 ± 5.41 c	14.2 ± 1.41 c	4.83 ± 1.14 b	6.82 ± 2.37 c	17.1 ± 4.10 c	33.0 ± 2.80 c	63.3 ± 7.05 c
BS2	82.7 ± 0.22 d	4.09 ± 0.10 d	-	1.39 ± 0.11 a	0.12 ± 0.00 d	11.8 ± 0.23 d	14.6 ± 0.15 d
G-BS2	64.2 ± 4.01 e	17.1 ± 2.41 c	4.57 ± 0.41 b	0.87 ± 0.09 b	2.12 ± 0.48 e	11.1 ± 1.45 e	21.3 ± 2.65 e
I-BS2	25.8 ± 3.93 c	16.7 ± 1.04 c	4.21 ± 0.47 b	5.26 ± 0.44 c	18.8 ± 1.75 c	29.2 ± 2.37 c	60.2 ± 3.70 c

Different letters within each column indicate a significant difference among the samples analyzed by one-way ANOVA followed by Tukey’s post-hoc test (*p* < 0.05). BS1 and BS2: black seed oils before digestion; G-BS1 and B-BS2: encapsulated black seed oil samples after gastric in vitro digestion; I-BS1 and I-BS2: encapsulated black seed oil samples after intestinal in vitro digestion; -: not detected.

**Table 2:** Abundances of different classes of oxylipins in black seed oil (BS1 and BS2) and lipid extracts of the digestates after gastric (G-BS1 and G-BS2) and intestinal (I-BS1 and I-BS2) in vitro digestion, expressed as the area under the peak of the corresponding MRM transition.

Oxylipins	BS1	G-BS1	I-BS1	BS2	G-BS2	I-BS2
HpODE	3.00 ± 0.57 a	61.6 ± 9.70 b	147 ± 28.8 c	4.47 ± 0.72 a	24.3 ± 8.02 d	85.6 ± 6.11 e
HODE	2.25 ± 0.26 a	27.8 ± 1.14 b	24.5 ± 1.98 c	2.32 ± 0.17 a	6.19 ± 0.83 d	5.82 ± 0.44 d
oxoODE	1.14 ± 0.10 a	10.6 ± 0.73 b	8.72 ± 0.77 c	1.06 ± 0.06 a	2.92 ± 0.39 d	2.71 ± 0.24 d
EpOME	0.42 ± 0.07 a	5.21 ± 0.22 b	4.48 ± 0.41 c	0.46 ± 0.05 a	1.21 ± 0.20 d	1.13 ± 0.09 d
DiHOME	2.67 ± 0.40 a	13.1 ± 1.47 b	9.23 ± 0.93 c	1.10 ± 0.10 d	1.83 ± 0.09 ad	1.19 ± 0.09 d
TriHOME	0.28 ± 0.09 a	5.89 ± 0.66 b	1.98 ± 0.36 c	0.25 ± 0.03 a	1.28 ± 0.14 d	0.63 ± 0.06 e
Total	9.76 ± 1.50 a	124 ± 13.9 b	196 ± 33.2 c	9.68 ± 1.13 a	37.7 ± 9.69 d	97.0 ± 7.04 e

Different letters within each row indicate a significant difference among the samples analyzed by one-way ANOVA followed by Tukey’s post-hoc test (*p* < 0.05). BS1 and BS2: black seed oils before digestion; G-BS1 and B-BS2: encapsulated black seed oil samples after gastric in vitro digestion; I-BS1 and I-BS2: encapsulated black seed oil samples after intestinal in vitro digestion.

**Table 3:** Concentration of tocols expressed in μg/g oil and of some sterols and thymoquinone given in molar percentage in relation to the total moles of AG+FA present in black seed oils (BS1 and BS2) and in the digestates after gastric (BS1-G and BS2-G) and intestinal (BS1-I and BS2-I) in vitro digestion.

Minor Compounds	BS1	G-BS1	I-BS1	BS2	G-BS2	I-BS2
α-Tocopherol (μg/g oil)	92.3 ± 3.19 a	64.7 ± 12.8 b	-	54.2 ± 9.74 b	18.8 ± 3.22 c	-
γ-Tocopherol (μg/g oil)	388 ± 77.4 a	318 ± 19.0 a	243 ± 26.0 b	112 ± 14.7 c	117 ± 8.72 c	37.2 ± 8.50 d
β-Tocotrienol (μg/g oil)	363 ± 12.8 a	326 ± 12.3 a	243 ± 29.2 b	394 ± 35.8 a	334 ± 17.6 a	226 ± 13.7 b
Δ7-Avenasterol (mmol/mol AG + FA)	0.10 ± 0.00 a	0.10 ± 0.01 a	0.07 ± 0.01 b	0.14 ± 0.01 c	0.14 ± 0.00 c	0.09 ± 0.01 d
Esters of cycloartenol (mmol/mol AG + FA)	0.29 ± 0.01 a	0.28 ± 0.01 a	0.27 ± 0.02 a	0.32 ± 0.02 a	0.28 ± 0.01 a	0.25 ± 0.02 a
Sitostanol (mmol/mol AG + FA)	0.05 ± 0.00 a	0.04 ± 0.01 a	-	0.05 ± 0.00 a	0.05 ± 0.00 a	-
Thymoquinone (mmol/mol AG + FA)	1.75 ± 0.01 a	1.26 ± 0.06 b	1.14 ± 0.06 c	2.77 ± 0.04 d	1.88 ± 0.11 e	1.66 ± 0.18 f

Different letters within each row indicate a significant difference among the samples analyzed by one-way ANOVA followed by Tukey’s post-hoc test (*p* < 0.05). BS1 and BS2: black seed oils before digestion; G-BS1 and B-BS2: encapsulated black seed oil samples after gastric in vitro digestion; I-BS1 and I-BS2: encapsulated black seed oil samples after intestinal in vitro digestion; -: not detected.

## Data Availability

Data are contained within the article or Supplementary Material.

## Supplementary Materials

The following supporting information can be downloaded at: https://www.mdpi.com/article/10.3390/antiox12010191/s1, composition of the juices employed in the in vitro digestion model, Table S1: Chemical shift assignments of ^1^H NMR signals of protons of acyl groups (AG), fatty acids (FA), and acylglycerols (MG: monoacylglycerols. DG: diacylglycerols. TG: triacylglycerols), alkanals and some sterols in CDCl3, Table S2: Free oxylipins detected by LC-MS based on previous publications [28,40], Figure S1: ^1^H NMR spectra of black seed oils 1 and 2 (BS1 and BS2) before the digestion, and the of the lipid extract from the digestates after gastric (G) and intestinal (I) in vitro digestion (G-BS1, I-BS1, G-BS2 and I-BS2), for the region between 3.6 and 4.4 ppm The signals are in agreement with those of Table S1.
