# Supplementary material for: Effect of Encapsulation Material on Lipid Bioaccessibility and Oxidation during In Vitro Digestion of Black Seed Oil
Source: Antioxidants (Basel). 2023 Jan 13;12(1):191. doi: 10.3390/antiox12010191 (PMC9854819; doi:10.3390/antiox12010191)
Supplement: Supplementary file 1 [file antioxidants-12-00191-s001.zip › antioxidants-2147152-supplementary.pdf]

Supplementary Material

Composition of the juices employed in the *in vitro* digestion model

- The saliva contained 12.02 mmol/L KCl, 5.10 mmol/L NaCl, 20.17 mmol/L $NaHCO_3$, 7.40 mmol/L NaH_2PO_4, 4.79 mmol/L Na_2SO_4, 2.06 mmol/L KSCN, 3.33 mmol/L urea, 0.09 mmol/L uric acid, 0.025 g/L mucin and 0.29 g/L A. oryzae α-amylase. The pH of the saliva was 6.9±0.1.
- The gastric juice contained 11.06 mmol/L KCl, 47.09 mmol/L NaCl, 0.22 mmol/L NaH_2PO_4, 5.72 mmol/L NH_4Cl, 2.72 mmol/L $CaCl_2*2H_2O$, 6.50 mL/L HCl (37 %), 1.42 mmol/L urea, 3.61 mmol/L glucose, 1.00 mmol/L bovine serum albumin, 3.00 g/L mucin, 100 U/mL A. niger lipase and 2.50 g/L pepsin. The pH was 1.3±0.1.
- The duodenal juice contained 7.57 mmol/L KCl, 119.98 mmol/L NaCl, 40.33 mmol/L $NaHCO_3$, 0.59 mmol/L KH_2PO_4, 0.53 mmol/l MgCl2, 1.36 mmol/L $CaCl_2*2H_2O$, 1.36 mL/L HCl (37 %), 0.18 mmol/L urea, 1.67 mmol/L glucose, 1.00 mmol/L bovine serum albumin, 9.00 g/L pancreatin and 1.5 g/L lipase type II from porcine pancreas. The pH of the duodenal juice was 8.1±0.1.
- The bile juice contained 5.05 mmol/L KCl, 89.99 mmol/L NaCl, 68.86 mmol/L $NaHCO_3$, 1.51 mmol/L $CaCl_2*2H_2O$, 0.15 mL/L HCl (37 %), 4.16 mmol/L urea, 1.80 mmol/L bovine serum albumin and 18.75 g/L bovine bile extract. The pH of the bile juice was 8.2±0.1.

Supplementary Table 1 (Table S1): Chemical shift assignments of ^{1}H NMR signals of protons of acyl groups (AG), fatty acids (FA), glycerides (MG: monoglycerides. DG: diglycerides. TG: triglycerides), alkanals and some sterols in $CDCl_3$.

Peak	Structures	Chemical shift (ppm)
**A**	AG in TG	2.36–2.26
	AG in 1,2-DG	2.33
	AG in 1,3-DG, 1-MG and FA	2.35
	AG in 2-MG	2.38
**B**	linoleic in AG and FA	2.77
**C**	linolenic in AG and FA	2.80
**D**	glyceryl group in **1-MG**	3.65
**E**	glyceryl group in **1,2-DG**	3.73
**F**	glyceryl group in **2-MG**	3.84
**G**	glyceryl group in **1-MG**	3.94
**H**	glyceryl group in **1,3-DG**	4.21-4.05
**I**	glyceryl group in **TG**	4.22
	**Oxidation compounds**	
	n-alkanals	9.75
	**Sterols**	
	esters of cycloartenol	0.58
	sitostanol	0.65
	Δ7-avenasterol	0.54
	thymoquinone	6.56-6.60

*The assignment of the ^{1}H NMR signals of the protons was done as in previous studies (Guillén & Ruiz, 2003; Ruiz-Aracama et al., 2017; Alberdi-Cedeño et al., 2020d; Goryainov et al., 2020).

Supplementary Table 2 (Table S2). Free oxylipins detected by LC-MS based on previous publications (Dufour & Loonis, 2005; Emami et al., 2020).

Free oxylipin	Precursor ion (m/z)	Product ion (m/z)	Collision energy (V)	Retention time (min)
**9(10)-EpOME**	295.3	171.1	7	25.0
**12 (13)-EpOME**	295.3	195.2	7	24.8
**9,10-DiHOME**	313.2	201.2	16	17.5
**12,13-DiHOME**	313.2	183.2	16	16.6
**9-HODE**	295.2	171.1	10	24.9
**13-HODE**	295.2	195.2	13	24.8
**9-oxo-ODE**	293.2	185.1	13	26.7
**13-oxo-ODE**	293.2	195.1	13	24.8
**9,10,13-TriHOME**	329.2	171.1	16	2.0
**9,12,13-TriHOME**	329.2	211.1	16	1.9
**HpOME**	311.1	293.2	10	25.8
**13-HpOME**	311.1	113.1	20	25.8
**9-HpOME**	311.1	123.0	20	25.8

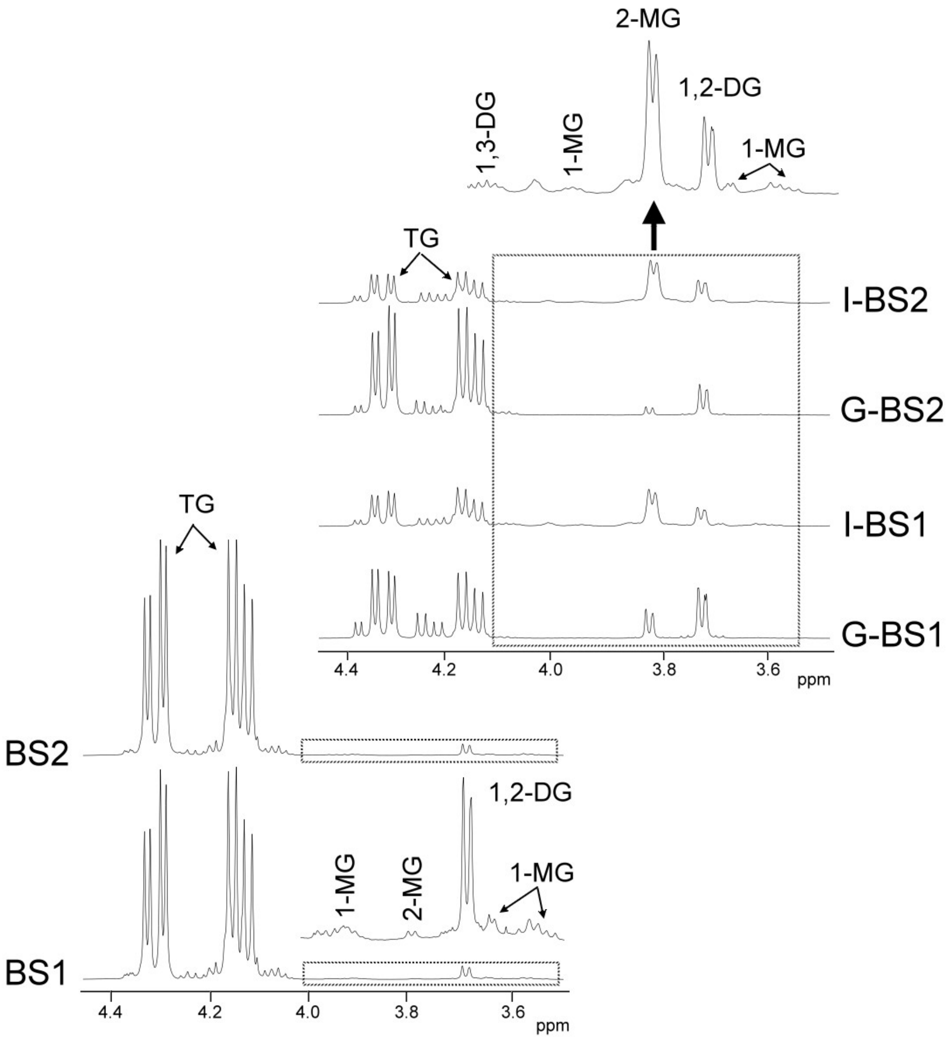

Supplementary Figure 1 (Figure S1). ^{1}H NMR spectra of black seed oils 1 and 2 (BS1 and BS2) before the digestion, and of the lipid extract from the digestates after gastric (G) and intestinal (I) *in vitro* digestion (G-BS1, I-BS1, G-BS2 and I-BS2), for the region between 3.6 and 4.4 ppm. The signals are in agreement with those of table S1.
